# Analysis of the Mitochondrial Genome in *Hypomyces aurantius* Reveals a Novel Twintron Complex in Fungi

**DOI:** 10.3390/ijms17071049

**Published:** 2016-06-30

**Authors:** Youjin Deng, Qihui Zhang, Ray Ming, Longji Lin, Xiangzhi Lin, Yiying Lin, Xiao Li, Baogui Xie, Zhiqiang Wen

**Affiliations:** 1 Mycological Research Center, College of Life Sciences, Fujian Agriculture and Forestry University, Fuzhou 350002, China; dengyoujin1980@163.com (Y.D.); llj_739@yahoo.com (L.L.); lxz0217xz@yahoo.com (X.L.); yy130816@yahoo.com (Y.L.); nevereverveer123@126.com (X.L.); mrcfafu@163.com (B.X.); 2 Center for Genomics and Biotechnology, Haixia Institute of Science and Technology, Fujian Agriculture and Forestry University, Fuzhou 350002, China; 3 Gutian Edible Fungal Research and Development Center, Ningde 352200, China; zz23161@163.com (Q.Z.); rming@life.illinois.edu (R.M.)

**Keywords:** cobweb disease, ORF, intron, homing endonuclease

## Abstract

*Hypomyces aurantius* is a mycoparasite that causes cobweb disease, a most serious disease of cultivated mushrooms. Intra-species identification is vital for disease control, however the lack of genomic data makes development of molecular markers challenging. Small size, high copy number, and high mutation rate of fungal mitochondrial genome makes it a good candidate for intra and inter species differentiation. In this study, the mitochondrial genome of *H.* H.a0001 was determined from genomic DNA using Illumina sequencing. The roughly 72 kb genome shows all major features found in other *Hypocreales*: 14 common protein genes, large and small subunit rRNAs genes and 27 tRNAs genes. Gene arrangement comparison showed conserved gene orders in *Hypocreales* mitochondria are relatively conserved, with the exception of *Acremonium chrysogenum* and *Acremonium implicatum*. Mitochondrial genome comparison also revealed that intron length primarily contributes to mitogenome size variation. Seventeen introns were detected in six conserved genes: five in *cox*1, four in *rnl*, three in *cob*, two each in *atp6* and *cox3*, and one in *cox2*. Four introns were found to contain two introns or open reading frames: cox3-i2 is a twintron containing two group IA type introns; cox2-i1 is a group IB intron encoding two homing endonucleases; and cox1-i4 and cox1-i3 both contain two open reading frame (ORFs). Analyses combining secondary intronic structures, insertion sites, and similarities of homing endonuclease genes reveal two group IA introns arranged side by side within cox3-i2. Mitochondrial data for *H. aurantius* provides the basis for further studies relating to population genetics and species identification.

## 1. Introduction

Cobweb disease is one of the most serious diseases in mushroom cultivation, affecting both mushroom quality and yield [1]. The disease has been found in many mushroom-growing countries, and affects many different types of important mushroom, including *Agaricusbisporus* [2,3], *Flammulina velutipes* [4], oyster mushroom [5], and *Hypsizygus marmoreus* [6], among others. Several species of *Cladobotryum* have been reported to cause cobweb disease, including *C. dendroides*, *C. mycophilum*, *C. varium*, *C. multiseptatum* and *C. verticillatum* [7,8]. Current research on identification of pathogens causing cobweb disease mainly focuses on symptoms, morphological features of the mycelium and conidium, as well as genetic characteristics of the internal transcribed spacer (ITS) region plus a partial 28S rDNA [6],which are insufficient for differentiation of intra-specific strains. As a result, it is difficult to determine whether different strains of the same pathogen have different host ranges, a feature which is important for design of proper control-measures.

The mitochondrial genome (mitogenome) has been used as a valuable tool in evolutionary biology and systematic studies, due to its small size, high copy number, and high mutation rate for easy detection [9,10,11]. As a cytoplasmic genetic material, the fungal mitogenome usually contains 14 conserved protein-coding genes, including three F0 subunits of the ATP-synthase complex, seven subunits of electron transport complex I, one subunit of complex III, and three subunits of complex IV, as well as genes for the large and small ribosomal RNAs, and a set of tRNA genes [12]. Some of these genes, such as *cox1* [13,14] and *cob* [15], have been used as DNA barcodes for species-level identification. On the other hand, the mitogenome size for fungi varies greatly, even for closely related species [9,16,17] and intra-specific strains [17], ranging from about 19 kbp for *Hanseniaspora uvarum* to more than 235 kbp for *Rhizoctonia solani* [18,19]. The number and length of introns are the most predominant factors contributing to the size differences of fungal mitochondria [9].

Different from nuclear genes, large introns are prevalent in fungal mitochondria, with the length mainly ranging from 150 bp to 4 kbp [20]. These introns are mainly classified into group I or group II according to their secondary RNA structures [21]. Group I introns usually carry a homing endonuclease gene (HEG), having the functions of transfer and site-specific integration of the intron [22]. These HEGs drive introns to distribute widely throughout horizontal DNA transfer, even between species with great phylogenetic distances [23]. Interestingly, a foreign intron may insert into another intron to form a particular intron-within-intron structure, known as twintron [24]. Accumulation of intron movement, as well as their degeneration, leads to intron polymorphism between closely related species, even in intra-specific level. The relatively high polymorphism and conserved flanking regions makes introns of fungal mitochondria ideal candidates for population genetic studies.

In this study, we determined the complete mitochondrial sequence of *Hypomyces aurantius* (teleomorph name *Cladobotryum variospermum*), a mycoparasite that causes cobweb disease. We described the gene content and genomic organization of the mycoparasite, as well as performed a comparative analysis of the known mitogenomes of *Hypocreales* fungi. Our focus was on mobile genetic elements such as group I introns and mobile ORFs. In addition, the potential formation of twintrons and introns with two HEGs is discussed. This study provides the basis for further research on population genetics and species identification of *H. aurantius*.

## 2. Results

### 2.1. Gene Content of the Mitogenome in H. aurantius

**Figure 1:**
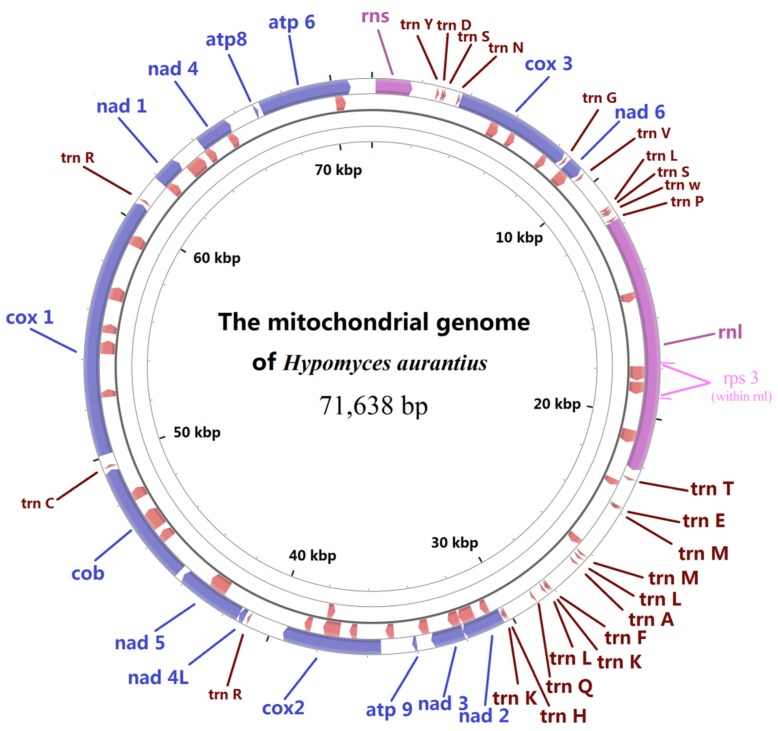
Physical circular map of the mitochondrial genome of *Hypomyces aurantius* H.a0001. The first nt of the *rns* gene was defined as nt 1 of the genome artificially. From outside to inside, the first ring represents genes encoding core proteins (blue), rRNAs (pink), and tRNAs (red); and the second ring represents the predicted ORFs (red) with clockwise direction; third ring represents the predicted ORFs (red) with anticlockwise direction. The name of each gene is indicated in its central region. The physical map was drawn using the program CG View [25].

The mitochondrial genome of *H. aurantius* H.a0001 (access number: KU666552) was extracted from the whole genome sequence obtained by de novo Illumina sequencing of total DNA. The final assembly resulted in a scaffold of 71,638 bp, representing a circular molecule with a GC content of 28.3%. Similar to most other known mitochondrial DNA (mtDNA) in ascomycetes, this genome contained 14 typical ascomycete protein-coding genes (Figure 1 and Table S1), which encoded three F0 subunits of the ATP-synthase complex (*atp6*, *atp8*, and *atp9*), seven subunits of electron transport complex I (*nad1*, *nad2*, *nad3*, *nad4*, *nad4L*, *nad5*, and *nad6*), one subunit of complex III (*cob*), and three subunits of complex IV (*cox1*, *cox2*, and *cox3*), as well as a gene coding the 40S ribosomal protein S3 (*rps3*, within the third intron of *rnl*). Additionally, 29 other conserved RNA genes were found in the genome, including 27 tRNA genes, and genes for large and small ribosomal RNA (*rnl* and *rns*).

In addition to the conserved genes, 30 ORFs were detected in the mitogenome, including 22 located within introns, and eight intergenic ORFs. Among intronic ORFs, three were found in each of *cob*, *cox2* and *cox3*, seven in *cox1*, four in *rnl*, and two in *atp6*. ORF102 and ORF105, both found in intron of *cox2* (cox2-i1), were located at opposite sides of ORF591, respectively. A BLASTX search showed that ORF591 was a LAGLIDADG_1 like gene, while ORF102 and ORF105, as well as their flanking sequences, belonged to aGIY-YIG encoding gene that was most similar to a GIY-YIG type homing endonuclease gene in the intron of *cox2* in *Ganoderma meredithae* (72% identity, accession number: YP_009129944.1), suggesting that ORF102 and ORF105 were not intact protein-coding genes, but rather parts of a degenerated GIY-YIG endonuclease gene. Sixteen other intronic ORFs were predicted to encode homologs belonging to homing endonucleases of LAGLIDADG (8 ORFs) orGIY-YIG (8 ORFs) families (Table 1). Mobile genetic elements were found not only in introns, but also in intergenic areas. Five out of eight intergenic ORFs were found to encode proteins similar to homing endonucleases.

One ORF with 1356 bp in length was detected in the area between *nad2* and *nad3* gene. BLASTX search showed the 1356-bp ORF consisted of a *nad3* gene and a laglidadg homing endonuclease-like fragment. The *nad3*-like fragment was most similar to *nad3* of *Ceratocystis cacaofunesta* 70% identify); and the laglidadg homing endonuclease-like fragment showed similarity to homing endonuclease in ribosomal protein 3/homing endonuclease-like fusion gene in *Sporothrix* sp. WIN(M)924 (46% identity).

### 2.2. Genetic Code

*H. aurantius* mitochondrial DNA was translated utilizing genetic code 4. All conserved protein-coding genes started with the canonical translation initiation codon (AUG), and ended with UAA, with the exception of *rps3* and *atp9*, which had termination codon of UAG (Table S1). Among 30 ORFs, nine started with non-canonical initiation codon, including ORF301 (AAC), ORF422 (UUA), ORF357 (CAA), ORF365 (AAA), ORF309 (ACU), ORF330 (AGU), ORF102 (AAA), ORF287 (UAC), and ORF322 (UAU). Twenty-one ORFs ended with UAA, and the terminal codons of the remaining nine were UAG. All of the conserved genes and ORFs were transcribed in the same direction with exception of one ORF in *cox2* (Figure 1).

The frequency of utilization of the 64 standard codons is shown in Table S2. UAA (encoding *Leu*) was the most frequently used codon, followed by AAA (*Lys*), AAU (*Asn*), AUA (*Lle*), UUU (*Ph*e), UAU (*Tyr*), GAA (*Glu*), AUU (*Ile*), GAU (*Asp*), and UCU (*Ser*). These 10 codons accounted for 48% of all codons, and the third base pair for all of these was either A or U. In addition, optimal codons for all amino acids ended either in A or U, with the exception of Met, which has a single codon (AUG). Both results were consistent with the high AT content of *H. aurantius* mitochondrial DNA (71.7%).

### 2.3. Phylogenetic Relationship Analysis

**Figure 2:**
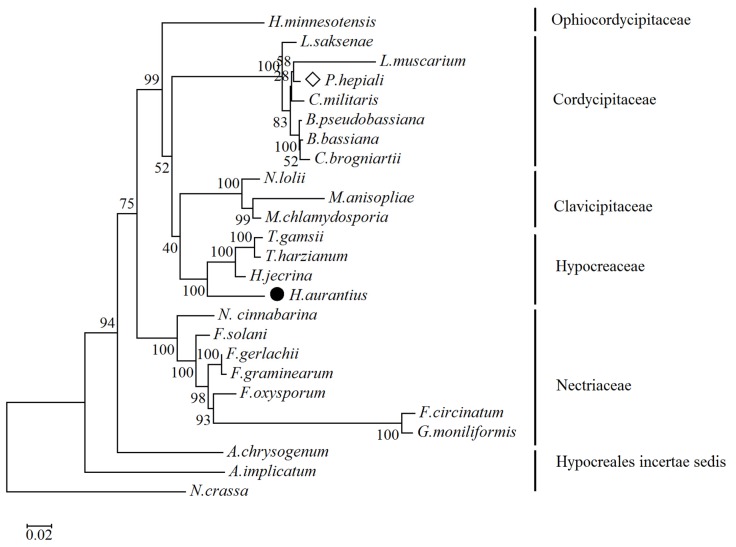
Phylogenetic tree of 24 mitochondrial proteomes in *Hypocreales*. A data set of 14 proteins was used, and topology was inferred using the Maximum Likelihood method (1000 replicates). Numbers above the nodes indicate bootstrap support values. Twenty-four mitochondria belong to the species of *A. chrysogenum* (NC_023268), *A. implicatum* (NC_026534), *Beauveria bassiana* (NC_010652), *Beauveria pseudobassiana* (NC_022708), *Cordyceps brongniartii* (NC_011194), *Cordyceps militaris* (NC_022834), *F. circinatum* (NC_022681), *Fusarium gerlachii* (NC_025928), *F. graminearum* (NC_009493), *Fusarium oxysporum* (AY945289), *F. solani* (NC_016680), *Gibberella moniliformis* (NC_016687), *Hirsutella minnesotensis* (NC_027660), *Hypocrea jecorina* (NC_003388), *Lecanicillium muscarium* (NC_004514), *Lecanicillium saksenae* (NC_028330), *Metarhizium anisopliae* (NC_008068), *Metacordyceps chlamydosporia* (NC_022835), *Neotyphodium lolii* (KF906135), *Nectria cinnabarina* (KT731105), *Paecilomyces hepiali* (diamond, KJ764671), *T. harzianum* (KR952346), *Trichoderma gamsii* (KU687109) and *H. aurantius* (filled circle, KU666552).

Utilizing existing fungal mitochondrial genomes as well as the novel *H. aurantius* mitogenome, the phylogenetic relationship in 24 species in *Hypocreales* was inferred, using *Neurospora crassa* as an outgroup. A phylogenetic tree was constructed based on 14 conserved protein-coding sequences, and a clear taxonomical relationship was shown (Figure 2). Twenty-four species of *Hypocreales* were clustered into six groups, mainly corresponding to the families *Ophiocordycipitaceae*, *Cordycipitaceae*, *Clavicipitaceae*, *Hypocreaceae*, *Nectriaceae*, and *Hypocreales incertae sedis*, respectively. As an exception, *P. hepiali* was clustered into *Cordycipitaceae*, and showed maximum similarity with *L. muscarium*, with a bootstrap value of 58%, despite belonging to the family *Clavicipitaceae* according to the NCBI taxonomy. *H. aurantius* was most closely related to *Trichoderma gamsii, Trichoderma. harzianum* and *Hypocrea jecorina*, all belonging to *Hypocreaceae*, with bootstrap value of 100%. The phylogenetic tree also indicated the genetic relationship between *A. chrysogenum* and *A. implicatum* is greater than that among other five families (*Ophiocordycipitaceae*, *Cordycipitaceae*, *Clavicipitaceae*, *Hypocreaceae* and *Nectriaceae*).

### 2.4. tRNA Gene Distribution and Gene Order Comparison in Hypocreales

**Figure 3:**
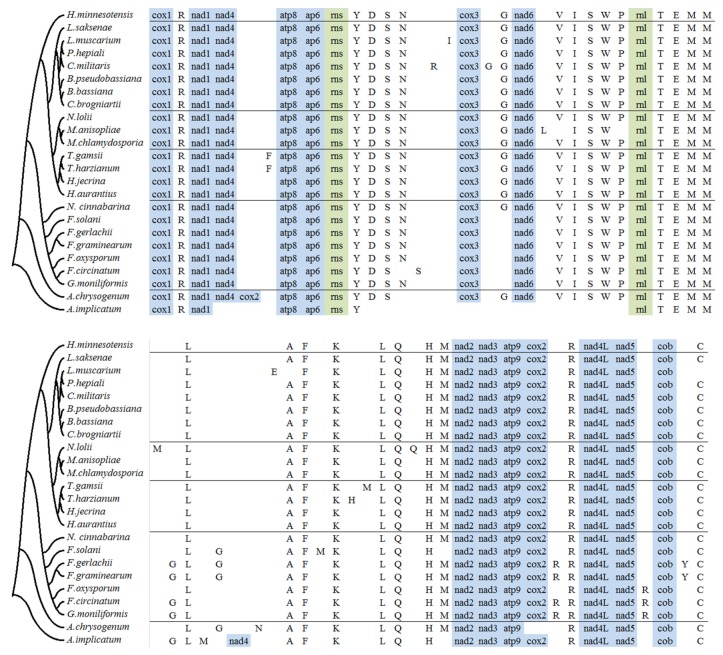
tRNA gene distribution and gene order comparison of 24 mitochondrial genomes in *Hypocreales*. Conserved protein coding genes (blue), rRNA (olive green) and tRNA genes were used. tRNAs with unknown function were not included. The fungi compared here were corresponding to 24 species used for phylogenetic tree construction. tRNA genes were indicated by their abbreviated letters.

The number of tRNA gene in 24 *Hypocreales* mitochondria mainly ranged from 24 to 28, with an exception of mitogenome in *A. implicatum*. Most of the tRNA genes were clustered into three groups, which located in regions between *rns* and *cox3* (YDSN), *nad6* and *rnl*(VISWP), and *rnl* and *nad2* (TEMMLAFKLQHM). Four tRNA genes were commonly found in these mitogenomes, but mainly scattered as a single gene, which were *trnR* between *cox1* and *nad1*, *trnG* between *cox3* and *nad6*, *trnR* between *cox2* and *nad4L*, and *trnC* between *cob* and *cox1.trnF* between *nad4* and *atp8* was detected only in two *Trichoderma* species (*T. gamsii* and *T. harzianum*); *trnG* between *cox3* and *nad6* was not found in only six *Fusarium* species. Besides, some tRNA gene gain and loss were taken place in one or few species. For example, *trnC* between *cob* and *cox1* lost in *L. muscarium*; *trnY* between *cob* and *cox1* gained in *F. gerlachii* and *F. graminearum* (Figure 3). Twenty-seven tRNA genes were identified in *H. aurantius*, two of which were detected to be function-unknown by tRNAscan-SE 1.3.1. Other 25 tRNA genes corresponded to all 20 standard amino acids. With the exception of *Met* (three copies), and *Leu*, *Arg* and *Ser* (two copies), only a single copy of the tRNA was found for each of the remaining 16 standard amino acids. Only 17 tRNA genes were found in the mitogenome of *A. implicatum*, which lost a fragment containing a set of tRNA genes (Figure 3).

Previously, the gene orders of 12 Hypocreales mitochondrial genomes were compared based on the arrangement of common protein genes, rRNA and tRNA genes [26]. Results showed that the gene order is essentially identical in these fungi. This previous research has been extended by this study, which has provided data on the order of conserved genes in the mitochondrial genomes of 12 species. As shown in the Figure, all tested species, with the exception of *A. chrysogenum* and *A. implicatum*, showed synteny in the gene order of conserved genes. Genome rearrangements took place in *A. chrysogenum* and *A. implicatum*, both belonging to the genus *Acremonium* of *Hypocreales incertae sedis*. In *A. chrysogenum*, *cox2* was found to be located in the area between *nad4* and *atp8*, while it was found in the sequence between *atp9* and *trnR* in the group of other 22 species. It was determined that *nad4*, ordered according to *nad1*-*nad4*-*atp8* in the group of other 22 species, was instead ordered according to *TEMMGLM*-*nad4*-*AFKLQH* in *A. implicatum* (Figure 3). In addition, two genes (*cox3* and *nad6*) were not found in the mitochondrial genome of *A. implicatum*.

### 2.5. Analysis of Mitogenome Size Variation

**Figure 4:**
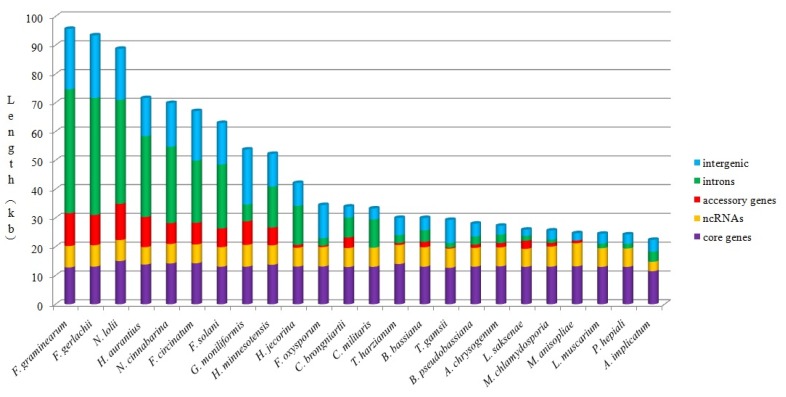
Contributions from intergenic regions, introns, ncRNAs, and core and accessory genes to mitochondrial genome sizes. Blue, green, red, yellow, and purple pillars represent the length of 14 core genes, introns, accessory genes, ncRNAs, and core genes, respectively. Each vertical pillar represents the size of a mitochondrial genome.

The mitogenome sizes of the fungi in *Hypocreales* varied drastically, ranging from 24,245 bp for *P. hepiali* to 95,676 bp for *F. graminearum*. To identify variation in genome size in mitochondria of *Hypocreales*, 23 additional mitogenomes (the same as those used for phylogenetic relationship analysis and gene order comparison) were used for comparative analysis. The length and number of introns in protein-coding genes and large and small subunit rRNA genes, as well as the length of core genes, non-coding RNAs (ncRNAs), accessory genes, and intergenic regions in each mitochondrial genome were calculated (Figure 4 and Table 2). Although there was great variation in mitochondrial genome sizes between the mitogenomes studied, the number and length of core genes and ncRNAs were relatively stable. Following *F. graminearum* (95.7 kb), *F. gerlachii* (93.4 kb) and *N. lolii* (88.8 kb), *H. aurantius* had the fourth largest mitogenome (71.7 kb), which contained the fourth largest size of overall amount of intronic DNA (28 kb). Mitogenome size was positively correlated with intron length (*r* = 0.975), which was the primary contributor of mitogenome size variation. Additionally, the length of intergenic regions and accessory genes were larger in most larger mitochondrial genomes, which indicated that these were important factors contributing to mitogenome length variation.

### 2.6. Introns in Conserved Genes

Six conserved genes in the mitogenome of *H. aurantius* were detected and found to contain a total of 17 introns, five of which were in *cox1*, four in *rnl*, three in *cob*, two each in *atp6* and *cox3*, and one in *cox2*. Intron-exon boundaries for the five genes were confirmed by comparing the corresponding intron-less sequences of *T. harzianum* (*cox1*, *cox2*, and *atp6*), *Metarhizium anisopliae* (*rnl*), and *H. jecorina* (*cox3*, Figure S1 [27]). The lengths of introns varied greatly, from 1128 bp for cox3-i1 to 3295 bp for cox2-i1, with an average length of 1799 bp. All introns were determined to belong to five subgroups of self-splicing Intron: IA, IB, IC1, IC2 and ID; no group II introns were found. Four large introns with sizes greater than 2300 bp (cox1-i3, cox1-i4, cox2-i1, and cox3-i2) contained two ORFs and/or two homing endonuclease motifs. Information relating to these introns, including position, size, type, ORFs, conserved domains, and best homologs is provided in Table 2.

**Figure 5:**
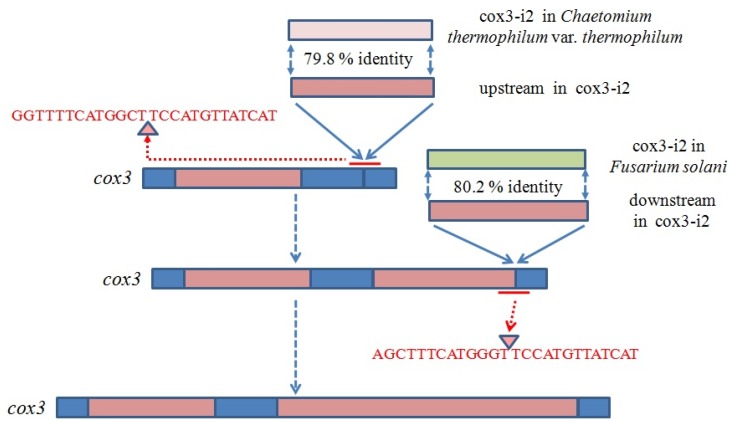
Probable forming process of cox3-i2. Blue regions represent exons, brown regions represent introns of *H. aurantius*, pink region represents cox3-i2 in *Chaetomium thermophilum* var. *thermophilum*, green region represents cox3-i2 in *Fusarium solani*, and red lines represent insertion sites in. Deduced insertion sites are shown in red font.

**Figure 6:**
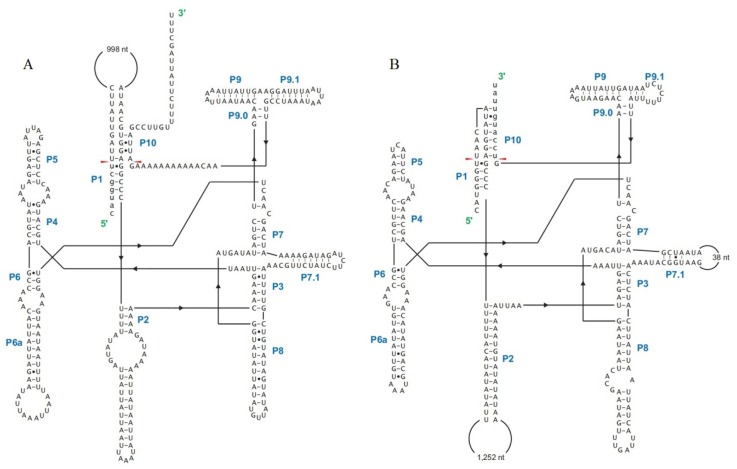
Predicted RNA secondary structures for upstream (**A**) and downstream introns (**B**) in cox3-i2. The 10 pairing regions (P1–P10) are indicated. The structure of the upstream intron is presented based on 1–1332 bp of cox3-i2 and seven base pairs of its upstream exon (lower cases in 5′ end of **A**); and the structure of the downstream intron is presented based on base pairs 1333–2887 of cox3-i2 and ten base pairs of its downstream exon (lower cases in 3′ end of **B**). Seven base pairs in the 5′ end of **B** (shown by lower case) are supposed to be a part of the upstream intron. The red arrows indicate the intron/“exon” junctions. The locations of corresponding homing endonuclease genes (HEG) are shown in the Figure. The HEG of upstream intron locates in its P1 loop; and that of downstream intron is in P2 loop. The models shown in the figure were assembled by combining the programs INFERNAL [29], Mfold [30], and Corel Draw™ (Corel Corporation: Ottawa, Canada).

**Figure 7:**
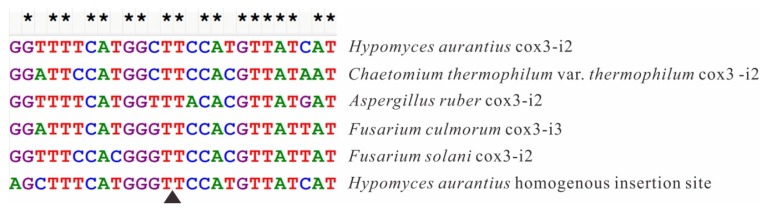
Comparison of 13-bp sequences upstream and downstream of insertion sites. Flanking sequences of cox3-i2 in *H. aurantius* were compared to best hits of the following ORFs: cox3-i2 in *C. thermophilum* var. *thermophilum*, cox3-i2 in *Aspergillusruber* CBS 135680, cox3-i3 in *Fusarium culmorum* strain CBS 139512, and cox3-i2 in *F. solani*, and the deduced later-inserted intron of cox3-i2 in *H. aurantius* was evaluated. Probable insertion site was shown by triangle; letters A, T, C, and G were shown by the color of green, red, blue, and brown, respectively. Asterisks represent base pairs are same in corresponding position. Sequences were aligned using Clustal W in MEGA 6.0.

Cox3-i2 was determined to be a complex intron consisting of 2877 bp, and containing two introns (Figure 5). Predicted RNA secondary structures showed that both introns were group IA1 (a subgroup of group IA) like introns, with upstream intron core structure consisting of 1–1332 bp of cox3-i2 and last 7 bp of its upstream exon, and downstream intron core structure containing 1333–2887 bp of cox3-i2 and first 10 bp of its downstream exon (Figure 6). Each double LAGLIDADG_1 motif was found in either introns: one located in the P1 loop of upstream intron, and the other was in P2 loop of downstream intron. No ORFs containing intact motifs were detected in both P1 loop of upstream and P2 loop of downstreams. BLASTN and alignment results revealed that upstream intron shared most similarity with cox3-i2 in *Chaetomium thermophilum* var. *thermophilum* (79.8% base pairs identity, Figure S2), while downstream intron was most similar to cox3-i2 in *F. solani* (80.2% base pairs identity, Figure S3). Both 13 bp of upstream and downstream of insertion site from two best hits of upstream intron (cox3-i2 in *C. thermophilum* var. *thermophilum* and cox3-i2 in *Aspergillusruber* CBS 135680) and downstream intron (cox3-i2 in *F. solani* and cox3-i3 in *F. culmorum*) in *H. aurantius*, together with that of cox3-i2 in *H. aurantius* were extracted for homology analysis using the Clustal W program in MEGA 6.0 [28]. Results showed that 18 out of 26 base pairs were identical, and seven differences were found in the third base pair of the triplet codon (Figure 7).

Cox2-i1 was the other complex intron in *H. aurantius*, with a size of 3295 bp. BLASTX analysis showed that this intron contained two separate fragments (base pairs 1–234 and 2418–3056), which concatenated to form a sequence encoding a GIY-YIG like motif. This sequence was most similar to the intronic ORF at intron of cox2 in *Ganoderma lucidum* (73% identity), *G. meredithae* (72% identity), and *A. bisporus* var. *burnettii* JB137-S8 (70% identity). These two separate fragments were interrupted by a 2183 bp fragment. No identified intron structure was found in the fragment, although an LAGLIDADG coding ORF was found.

Other than cox3-i2 and cox2-i1, two complex introns were identified: cox1-i4 and cox1-i3. Two ORFs were identified in the fourth intron of *cox1* (cox1-i4, 2334 bp), one of which encoded a homing endonuclease with a LAGLIDADG_1 motif most similar to an intronic ORF in intron 9 in *Ceratocystis cacaofunesta cox1* (79% identity), and the other of 1179 bp in length, not containing any conserved domains encoding any homologs. The cox1-i3 intron was found to be 2310 bp and contained two ORFs (ORF422 and ORF308), both encoding products of a GIY-YIG conserved domain.

## 3. Discussion

The present study was the first report of sequencing of the mitochondrial genome in cobweb disease. The complete mitochondrial genome of *H. aurantius* was sequenced using next-generation sequencing technologies. Similar to the mitogenomes of other fungi, the 71,638 bp mtDNA of *H. aurantius* contained two sets of sequences with great differences in the evolution rate. Fourteen protein-coding genes, including three F0 subunits of the ATP-synthase complex, seven subunits of electron transport complex I, one subunit of complex III, and three subunits of complex IV, were relatively conserved, and commonly found in 24 published mtDNA sequences of *Hypocreales*. A phylogenetic tree based on these genes mainly reflected the taxonomic status of these species, demonstrating that the fourteen genes could be used for interspecies analyses, including species identification and phylogenic analysis. On the other hand, the gain and loss of introns in these conserved genes, as well as genes for large and small subunit rRNAs, may be transferred via horizontal gene transfer [31], and often vary in number, size, gene content and insertion site among closely related species or even intra-species [32,33,34,35]. To demonstrate this, introns of six *Fusarium* species were compared in this study, *F.graminearum* contained 29 introns with total size of 43,056 bp, *F. gerlachii* had 28 introns of 40,672 bp, *F. solani* had 15 introns of 22,223 bp, *F. circinatum* had 16 introns of 21,648 bp, *G. moniliformis* contained four introns of 5826 bp total, and *F. oxysporum* contained only two introns of 2612 bp. The high evolution rate makes mitochondrial introns available to differentiate strains within the same genus or even intra-species.Seventeen large introns were detected in the mitogenome of *H. aurantius*, four out of which were found in large subunit rRNA, and 13 in protein-coding genes. Information pertaining to these introns, as well as their conserved flanking sequences, may serve as the basis for design of molecular markers for pathogenic differentiation in cobweb disease. In addition, the smaller size (~72 kb vs. 32 Mb for nuclear genome), high copy number (~8 copies), single-copy number of main conserved protein coding genes (with except of *nad3*, which is present two copies) make analyses utilizing molecular markers originating from *H. aurantius* mitochondria more convenient to perform.

Duplicated copies of conserved protein-coding genes are common in fungal mitochondrial genomes. MtDNA of *Sclerotinia borealis* was detected to contain a truncated extra copy of *atp9*, which consists of full-size *atp9* lacking a stop codon [36]. An inverted region of 6075 bp in size was found in mtDNA of *Phlebia radiata*, which harbored an additional copy of *atp6* and the gene encoding tRNA-Ile with only a three-nucleotide difference (whole inverted region) [37]. The duplication was also found in the mtDNAof *Phialocephala subalpine* [38] and *Botryotinia fuckeliana* [36]. *In the present study*, two *nad3*-like genes were detected in mtDNA of *H. aurantius*. BLASTX analysis showed that a 1356-bp *nad3*-like gene was most similar to the *nad3* gene of *C. cacaofunesta* (70% identify, different order than *H. aurantius*), while a 414 bp *nad3* gene was identified as a best match with that of *Hypocrea jecorina* (90% identity), which is most close to *H. aurantius* among species analyzed in the study (Figure 2). It is possible that the 414-bp *nad3*-like gene is more likely to be *nad3* in the mitochondrial genome. Further analysis revealed that the 1356-bp *nad3*-like gene contained a LAGLIDADG homing endonuclease like sequence near the 3′end.The homing endonuclease like sequence showed similarity to homing endonuclease in ribosomal protein 3/homing endonuclease-like fusion gene in *Sporothrix* sp. WIN(M)924 (46% identity), which is a complex gene-within-a gene created by an insertion of HEG into rps3 gene at the 3′-end [39]. It was suggested that the 1356 bp *nad3*-like gene was a *nad3*/homing endonuclease-like fusion gene. The fusion gene might result from a horizontal gene transfer event of a homing endonuclease gene near *nad3* of other fungi, which carried a fragment of *nad3* gene during the movement. The events may be an important way in conserved gene duplication of fungal mitochondria.

Pantou et al. [40] and Lin et al. [26] did the mitochondrial gene order comparison of members in *Hypocreales*, and obtained similar results: small differences were found in gene order based on the distribution of conserved protein-coding genes, tRNA genes and rRNAs. In this study, we extend our comparison to 24 mitogenomes. The results showed that gene order among *Hypocreales*, except for *A. chrysogenum* and *A. implicatum*, is relatively conserved, which was consistent with the finding of Pantou et al. and Lin et al. There are three rearrangement events taken place in *A. chrysogenum* or *A. implicatum*, both of which belong to *Hypocreales incertae sedis*. These results are corresponding to the result of their phylogenetic analyses, demonstrating that the genetic relationship between these two species is farther than that between any two species from five families of *Ophiocordycipitaceae*, *Cordycipitaceae*, *Clavicipitaceae*, *Hypocreaceae* and *Nectriacea*. Gene order variability in fungi may largely result from recombination [41]. In the present research, a fragment including *cox2* was found to move from the area between *atp9* and *nad4L* in 23 species to the area between *nad4* and *atp8* in *A. chrysogenum*. In addition, the sequence containing *nad4* changed location from the area of nad1-nad4-atp8 in 23 species to of TEMMGLM-nad4-AFKLQH in *A. implicatum*. Both events were likely due to recombination.

Twintrons (Twin introns) were first discovered by Donald W. Copertino and Richard B. Hallick in the *psbF* gene of *Euglena* chloroplast [24], and were thought to be formed by insertion of a mobile intron into an existing intron. The *psbF* twintron is a group II intron inserted into another group II intron. Since the initial discovery, many more twintron categories have been identified. The most common type is an intron inserted by another intron of the same group type [42,43]. Hafez et al. [44] discovered the mS1247 twintron in the mitochondrial *rns* gene, which was detected to be a group I intron interrupted by a group II intron. This type of twintron, containing two different groups of introns, was also found in the plastid genome of the cryptophyte *Rhodomonas salina* [45]. Moreover, complex twintrons were reported to contain multiple intron insertions into pre-existing introns [46], which indicates that twintrons may contain more than two introns. All twintrons described above are characterized by an intron interrupted by other introns. In the present study, cox3-i2 intron of *H. aurantius* was detected to be 2877 bp in size and to contain two group IA intron core structures using the RNAweasel algorithm. Sequence comparisons revealed that upstream part (position from 1 to 1318 bp) of cox3-i2 was most similar with cox3-i2 in *C. thermophilum* var. *thermophilum* (~80%, Figure S2), and the downstream part (position from 1340 to 2871 bp) matched well with cox3-i2 in *F. solani* (~80%, Figure S3), indicating fragment from 1 to 1318 bp and fragment from 1340 to 2871 bp belong to two different introns; Predicted RNA secondary structures revealed that upstream and downstream introns were arranged side by side. Previous studies revealed that high similar homing endonucleases share homogenous insertion sites [32,47,48,49]. Phylogenetic analysis revealed HEG in upstream intron showed highest similarity with ORF330 in cox3-i2 of *C. themophilum* var. *thermophilum* and ORF338 in cox3-i2 of *A*. *ruber*, and HEG in downstream intron matched well with ORF426 in cox3-i2 of *F*. *solani* and ORF436 in cox3-i3 of *F*. *culmorum* (Figure S4). The 13-bp flanked sequences of insertion sites of putative arrangements were compared with that of their best hits. Results revealed the most possible formation of the cox3-i2 intron was to be as follows, an intron containing 1339 bp (upstream intron, ending with AGCTTTCATGGGT) was initially inserted into *cox3*, forming a new homogenous insertion site at the 3′ end of the intron. The 1538-bp intron (downstream intron) was then inserted into this site to form the twintron with two introns in tandem array (Figure 5).

The terminal sequence of the upstream intron might serve as a “pseudo-exon” providing the sequence required for the downstream intron to assemble the P1 and P10 interactions. Thus, a splicing competent RNA fold can be formed allowing the downstream intron to splice first followed by the upstream intron assembling itself into a configuration that permits for auto splicing to proceed. To our knowledge, this is the first report of a twintron with two intact introns arranged side by side in fungi.

Cox2-i1, the other complex intron identified in *H. aurantius*, is a group IB type intron, encoding two HEs. The GIY-YIG-like HE is thought to have been interrupted by a fragment with ORF591 encoding a LAGLIDADG like-HE from positions 235 2417 bp. The GIY-YIG-like HE was most similar to the intronic ORF in an intron of *cox2* in *G. lucidum* (73% identify), *G. meredithae* (72% identify), and *A. bisporus* var. *burnettii* JB137-S8 (70% identify). All of these, together with cox2-i1 were found to share homogenous insertion sites. It therefore seems that the interrupted HE was carried by former intron insertion, while the LAGLIDADG-like HE was obtained from later intron insertion. With the exception of ORF591, the interrupting fragment contained sequences of 248bp and 158bp at the 5′ and 3′ ends; no identified intron structure was detected in these regions. Previous studies [50,51] showed that nadl-i4 and coxl-i7 introns in *Podospora anserina* mitochondrial genomes can be monorfic, having a single ORF, or biorfic, having an optional ORF together with a fragment (292 bp for nad1-i4, 49 bp for cox3-i7) downstream of its stop codon transfer to monorfic intron to form a biorfic intron. The formation of the Cox2-i1 intron of *H. aurantius* may be similar to biorfic introns nadl-i4 and coxl-i7. Orf591 functioned as a mobile element to transfer into a former intron to form a biorfic intron.

## 4. Materials and Methods

### 4.1. Fungal Strains and DNA Preparation

*H. aurantius* H.a0001 was obtained from the Edible Fungal Germplasm Resources Management Center of Fujian province, Fuzhou, China. After culture in potato dextrose broth at 25 °C and dark condition for seven to eight days, mycelial mats of *H. aurantius* H.a0001 were harvested, and washed three times with sterile deionized water and freeze-dried. Whole genomic DNA extraction was performed using the CTAB method as previously described by Manicon et al. [52].

### 4.2. Genome Sequencing and Mitochondrial DNA Assembly

*H. aurantius* genomic DNA (~3 μg) was used to construct shotgun libraries following the Illumina sequencing protocol. Whole genome sequencing was performed in a HiSeq 2500 Illumina sequencer (Novogene Bioinformatics Institute, Beijing, China) to obtain 2 × 100 bp reads with insertion sizes of ~500 bp. All reads (20,098,455 reads, 4.02 Gbp) were used to assemble using the software Velvet 1.2.03 [53] with different Kmer values. Mitochondrial contigs were picked out from assembly result by comparing with mitochondrial DNA of other species in *Hypocreales* using BLASTX. Mitochondrial contigs with different Kmers were placed in proper order, resulting in a single circular mtDNA 71,638 bp in size.

### 4.3. Gene Annotation and Bioinformatic Analysis

Protein-coding and rRNA genes were annotated by the program Mfannot version 1.33 [54]). tRNA genes were identified by the program tRNAscan-SE 1.31 with tRNA Cove cutoff score of 15. Orfs with size greater than 300 bp were detected using the same program and the Mold, Protozoan, and Coelenterate Mitochondrial Code and the Mycoplasma/Spiroplasma Code (NCBI translation table 4). Intron-exon boundaries in conserved genes were identified manually by ClustalX [55] based on comparisons with corresponding intron-less sequences of other known mitogenomes. HEGs were recognized by BLASTX queries against the non-redundant NCBI database. Intron types were determined with RNAweal algorithm [31]. The physical map of the *H. aurantius* mitogenome was generated using the program CG view [25]. The RNA secondary structures of the cox3-i2 intron (the second intron of *cox3*) were predicted using the program INFERNAL [29] by aligning with published group I introns [44,56,57,58]. Structure alignment data for most existing group I introns was available in GISSD (Group I intron structural alignment database) [59]. Key stem-loop structure elements were identified using the online program Mfold [30], and final secondary structures were drawn using CorelDraw™ software.

### 4.4. Gene Order and Phylogenetic Analysis

Gene order of fourteen conserved protein-coding genes, tRNA genes as well as large and small ribosomal RNA genes, in *H. aurantius* was compared with that of 23 other known mitochondria in *Hypocreales*, including *A. chrysogenum* (NC_023268), *A. implicatum* (NC_026534), *B. bassiana* (NC_010652), *B. pseudobassiana* (NC_022708), *C. brongniartii* (NC_011194), *C. militaris* (NC_022834), *F. circinatum* (NC_022681), *F. gerlachii* (NC_025928), *F. graminearum* (NC_009493), *F. oxysporum* (AY945289), *F. solani* (NC_016680), *G. moniliformis* (NC_016687), *H. minnesotensis* (NC_027660), *H. jecorina* (NC_003388), *L. muscarium* (NC_004514), *L. saksenae* (NC_028330), *M. anisopliae* (NC_008068), *M. chlamydosporia* (NC_022835), *N. lolii* (KF906135), *N. cinnabarina* (KT731105), *P. hepiali* (KJ764671), *T. harzianum* (KR952346), and *T. gamsii* (KU687109). The fourteen proteins included three ATPase subunits (*atp6*, *atp8*, and *atp9*), four subunits of the respiratory chain complexes (*cox1*, *cox2*, *cox3*, and *cob*), and seven NADH dehydrogenase subunits (*nad1*, *nad2*, *nad3*, *nad4*, *nad4L*, *nad5*, and *nad6*). The concatenated sequences of these fourteen proteins were also used for phylogenetic analysis. With the exception of seventeen *Hypocreales* species described above, *N. crassa* was included to construct phylogenetic trees as an outgroup. Sequence alignment was performed using the Clustal W program of the MEGA 6.0 [28] package. Gap opening penalty and extension penalty for both pairwise alignment and multiple alignment were set to 10 and 3, respectively. Phylogenetic trees were inferred using the Maximum Likelihood method using the MEGA 6.0 package. Boostrap values were determined using 1000 replicates.

## Figures and Tables

**Table 1:** Information of introns.

Gene	Intron	Position	Intron Size, bp	Intron Type	ORF	Conserved Domain	*E*-Value	Id	Similarity	Accession
*cox3*	Intron 1	73 aa	1128	IB	ORF309	LAGLIDADG_1	1.00 × 10^−172^	79%	*Ceratocystis cacaofunesta*	YP_007507087
	Intron 2	213 aa	2877	IA(5’)	degraded ORF	LAGLIDADG_1	9 × 10^−119^	59%	*Annulohypoxylon stygium*	YP_008964946
				IA	degraded ORF	LAGLIDADG_1	0	80%	*Fusarium solani*	YP_005088126
*cox2*	Intron 1	76 aa	3295	IB	*	GIY-YIG	1.00 × 10^−92^	73%	*Ganoderma lucidum*	CCQ18569
					ORF591	LAGLIDADG_1	6.00 × 10^−78^	46%	*Podospora anserina*	CAA38805
*cob*	Intron 1	67 aa	1586	IB(3’)	ORF453	LAGLIDADG_1	0	90%	*Sordaria macrospora k-hell*	XP_003342387
	Intron 2	131 aa	1202	ID	ORF287	GIY-YIG	7.00 × 10^−144^	79%	*Fusarium acuminatum*	CDL73465
	Intron 3	169 aa	1069	IB(3’)	ORF322	LAGLIDADG_1	0	83%	*Aspergillus nidulans*	P03880
*rnl*	Intron 1	622 bp	1790	IC1	ORF308	GIY-YIG	8.00 × 10^−77^	74%	*Sclerotinia borealis*	YP_009072316
	Intron 2	811 bp	1666	IC1	ORF342	GIY-YIG	3.00 × 10^−112^	70%	*Cordyceps brongniartii*	YP_002213592
	Intron 3	2494 bp	2090	IA	ORF474	rps3	0	82%	*Trichoderma harzianum*	AKK32420
	Intron 4	2599 bp	1852	IB(5’)	ORF294	GIY-YIG	5.00 × 10^−70^	49%	*Sclerotinia borealis*	YP_009072319
*cox1*	Intron 1	94 aa	1260	IB	ORF301	LAGLIDADG_1	5 × 10^−162^	80%	*Ganoderma meredithae*	YP_009129958
	Intron 2	180 aa	1385	IC2	ORF245	LAGLIDADG_1	‒	‒	*‒*	‒
	Intron 3	352 aa	2310	IB	ORF422	GIY-YIG	0	74%	*Madurella mycetomatis*	YP_006576207
					ORF308	GIY-YIG	2 × 10^−167^	83%	*Fusarium culmorum*	CDL73521
	Intron 4	375 aa	2334	IB	ORF357	LAGLIDADG_1	0	79%	*Ceratocystis cacaofunesta*	YP_007507075
					ORF392	‒	‒	‒	*‒*	‒
	Intron 5	427 aa	1759	IB	ORF447	GIY-YIG	0	75%	*Fusarium graminearum*	AKB93468
*atp6*	Intron 1	116 aa	1404	IB	ORF365	LAGLIDADG_1	1.00 × 10^−145^	71%	*Botrytis cinerea*	AGN49025
	Intron 2	189 aa	1584	IC2	ORF302	GIY-YIG	7.00 × 10^−133^	72%	*Podospora anserina*	NP_074919

Note: * indicates ORFs not detected because of degeneration; ‒ represents values that were not present or not observed, or unable to be calculated; Id, percent identity.

**Table 2:** Mitochondrial genomic comparison of 17 *Hypocreales* Species.

Genome ^a^	Mitogenome Size	GC %	Protein-Coding Genes	Protein-Coding Genes with Introns	Length (and Number) of Introns in Protein Coding Genes (bp)	Length (and Number) of Intron in Large Subunit rRNA (bp)	Length (and Number) of Intron in Small Subunit rRNA (bp)	tRNAs ^b^	Intronic ORFS	Length (and Number) of Non-Intronic Accessory Genes (bp)	Accession
*Acremonium chrysogenum*	27,266	26.54	19	1	1331 (2)	1602 (1)	0	26	2	1482 (3)	NC_023268
*Acremonium implicatum*	22,376	26.12	15	0	0	0	0	17	0	2556 (3)	NC_026534
*Beauveria bassiana*	29,961	27.25	20	2	2272 (2)	1745 (1)	0	25	2	1872 (3)	NC_010652
*Beauveria pseudobassiana*	28,006	27.54	17	1	1078 (1)	1755 (1)	0	25	2	1116 (1)	NC_022708
*Cordyceps brongniartii*	33,926	27.34	22	2	3776 (3)	3095 (2)	0	25	4	3663 (4)	NC_011194
*Cordyceps militaris*	33,277	26.79	22	4	4707 (4)	5116 (4)	0	27	7	0	NC_022834
*Fusarium circinatum*	67,109	31.45	33	5	19,269 (15)	2379 (1)	0	27	17	7506 (2)	NC_022681
*Fusarium gerlachii*	93,428	31.91	53	8	38,673 (27)	1999 (1)	0	28	31	10,479 (8)	NC_025928
*Fusarium graminearum*	95,676	31.84	53	8	41,057 (28)	1999 (1)	0	28	30	11,388 (9)	NC_009493
*Fusarium oxysporum*	34,477	30.98	17	1	1010 (1)	1602 (1)	0	25	2	456 (1)	AY945289
*Fusarium solani*	62,978	28.88	34	6	20,427 (14)	1796 (1)	0	25	14	6495 (2)	NC_016680
*Gibberella moniliformis*	53,753	32.61	24	2	3909 (3)	1953 (1)	0	27	4	8175 (2)	NC_016687
*Hirsutella minnesotensis*	52,245	28.42	30	4	12,428 (10)	1818 (1)	0	25	11	6145 (5)	NC_027660
*Hypocrea jecorina*	42,130	27.24	25	3	11,934 (9)	1655 (1)	0	26 ^b^	9	960 (2)	NC_003388
*Lecanicillium muscarium*	24,499	27.15	15	0	0	1617 (1)	0	25	1	0	NC_004514
*Lecanicillium saksenae*	25,919	26.53	17	0	0	1668 (1)	0	25	1	2829 (2)	NC_028330
*Metarhizium anisopliae*	24,673	28.40	15	0	0	0	0	24	0	924 (1)	NC_008068
*Metacordyceps chlamydosporia*	25,615	28.28	18	0	0	1147 (1)	0	25	1	1293 (2)	NC_022835
*Neotyphodium lolii*	88,756	27.53	60	7	30,040 (19)	4809 (4)	1288 (1)	28 ^b^	21	12,576 (22)	KF906135
*Nectria cinnabarina*	69,895	28.71	33	6	23,840 (15)	2762 (2)	0	25	11	7197 (8)	KT731105
*Paecilomyces hepiali*	24,245	26.64	15	0	0	1610 (1)	0	25	1	0	KJ764671
*Trichoderma harzianum*	29,999	27.78	17	1	1118 (1)	1631 (1)	0	27	2	639 (1)	KR952346
*Trichoderma gamsii*	29,303	28.25	16	0	0	1609 (1)	0	28 ^b^	1	348 (1)	KU687109
*Hypomyces aurantius*	71,638	28.31	42	5	20,136 (13)	7972 (4)	0	27 ^b^	18	10,482 (12)	KU666552

^a^ Genomes were annotated using MFannot; ^b^ means containing one or two function-unknown tRNAs.

## Supplementary Materials

Supplementary materials can be found at http://www.mdpi.com/1422-0067/17/7/1049/s1.
