# Supplementary material for: Analysis of the Mitochondrial Genome in Hypomyces aurantius Reveals a Novel Twintron Complex in Fungi
Source: Int J Mol Sci. 2016 Jun 30;17(7):1049. doi: 10.3390/ijms17071049 (PMC4964425; doi:10.3390/ijms17071049)
Supplement: Supplementary file 1 [file ijms-17-01049-s001.pdf]

Supplementary Materials: Analysis of the Mitochondrial Genome in *Hypomyces aurantius* Reveals a Novel Twintron Complex in Fungi

Youjin Deng, Qihui Zhang, Ray Ming, Longji Lin, Xiangzhi Lin, Yiying Lin, Xiao Li, Baogui Xie and Zhiqiang Wen

```
CLUSTAL O(1.2.1) multiple sequence alignment


Hypomyces_aurantius_cox3      ATGACAACTTTAACTAGAAGTAATTTTCAAGATCATCCTTTCCATTTAGTGTCACCTTCT
Hypocrea_jecorina_cox3        ATGACAACTTTAACTAGAAGTAATTTTCAAGATCATCCTTTCCATTTAGTGTCACCTTCA
                              *********************************************************** 

Hypomyces_aurantius_cox3      CCTTGACCTTTATACACAAGTATATCTTTATTTACTTTAACAGTAAATGGAGCATTATCT
Hypocrea_jecorina_cox3        CCGTGACCTTTATACACAAGTATATCTTTATTTAATTTAACTGTAAATGGAGCATTATCT
                              ** ******************************* ****** ******************

Hypomyces_aurantius_cox3      ATGCATCTATTCAGTAATAGCTATATATTATTCTTTATAGCTCTAGGTACAGTAGTAGCA
Hypocrea_jecorina_cox3        ATGCATCTTTTCAATAATAGCTATATATTCTTCTTTATAGCTTTAGCTACAGTAATTTCA
                              ******** **** *************** ************ *** ******* *  **
                                                            Cox3-i1 insertion site
Hypomyces_aurantius_cox3      TCAATGTCTTTATGATTCAGAGATATTATCTCTGAAGGT⇩ACATATTTAGGTAACCATACA
Hypocrea_jecorina_cox3        TCAATGGCTTTATGATTCAGAGACATAGTATCAGAAGGT ACATTTTTAGGTAATCACACT
                              ****** **************** **  * ** ****** **** ********* ** **

Hypomyces_aurantius_cox3      TTATCCGTTCAAAAAGGATTAAATTTAGGAGTTATATTATTTATAGTATCTGAAGCTTTA
Hypocrea_jecorina_cox3        CTTGCTGTTCAAAAAGGATTAAATTTAGGTGTAATATTATTTATAGTATCTGAAGCTTTA
                               *  * *********************** ** ***************************

Hypomyces_aurantius_cox3      TTCTTCTTAGCTATATTCTGAGCATTCTTTCATAGTGCTTTAACACCTACAGTTGAATTA
Hypocrea_jecorina_cox3        TTCTTCTTAGCTATATTCTGAGCATTCTTCCATAGTGCTTTAACACCTACAGTTGAATTA
                              ***************************** ******************************

Hypomyces_aurantius_cox3      GGTGCTCAATGACCACCTATGGGTATAGAACCTATAAATCCTTTCGAATTACCTTTATTA
Hypocrea_jecorina_cox3        GGTGCTCAATGACCACCTATGGGTATAGAACCTATAAATCCTTTTGAATTACCTTTATTA
                              ******************************************** ***************

Hypomyces_aurantius_cox3      AACACAGTTATATTGTTATCTAGTGGTGCTACAATTACTTATGCTCACCATTCTTTAATT
Hypocrea_jecorina_cox3        AACACAGTTATATTATTATCTAGTGGTGCTACAATTACTTTTGCTCATCATAGTTTAATA
                              ************** ************************* ****** ***  ****** 

Hypomyces_aurantius_cox3      AAAGGTGAAAGATCAGGAGCTTTATATGGTACTATTTCGACAGTAGTATTAGCTTTAGTA
Hypocrea_jecorina_cox3        AAAGGTGAAAGATCAGGAGCATTATATGGAACTATTTTCACAGTAGTATTAGCTTTAATA
                              ******************** ******** *******  ****************** **

Hypomyces_aurantius_cox3      TTTACTTTATTTCAAGGAGTAGAATATAGCGTTTCTTCATTTACAATAAGTGACGGTGTA
Hypocrea_jecorina_cox3        TTTACTTTATTCCAAGGAGTAGAATATAGCGTTTCTTCATTCACAATAAGTGACGGTGTA
                              *********** ***************************** ******************
                                                          Cox3-i2 insertion site
Hypomyces_aurantius_cox3      TTTGGAACATGTTTCTTCTTCGGAACAGGTTTTCATGGCT⇩TCCATGTTATCATAGGTACA
Hypocrea_jecorina_cox3        TTTGGAACATGTTTCTTCTTTGGAACAGGTTTCCACGGAT TCCACGTTATTATAGGTACT
                              ******************** *********** ** ** * **** ***** ******** 

Hypomyces_aurantius_cox3      ATCTTCTTATCTGTAGGATTATGAAGAATAATGGCATATCATTTAACAGATCATCACCAT
Hypocrea_jecorina_cox3        GTTTTCTTATCTGTTGCATTGTGAAGAATAATGGCATACCATTTAACAGATCATCATCAT
                               * *********** * *** ***************** ***************** ***

Hypomyces_aurantius_cox3      CTTGGTTATGAAGCTGGAATATTATACTGACATTTTGTAGATGTTGTTTGATTATTCTTA
Hypocrea_jecorina_cox3        CTTGGTTACGAAGCTGGAATATTATACTGACATTTTGTAGATGTTGTTTGATTATTCTTA
```

Figure S1. *Cox3* gene sequence alignment between *H. aurantius* and *H. jecorina*. Intron-less *cox3* gene sequence of *H. jecorina* was used to compare against that of *H. aurantius* to confirm locations of introns of cox3 gene in *H. aurantius.* Results showed that the second intron located in position of 640 bp and was 2,877 bp in size. No exon sequences was found in the large intron. Consensus sequences were marked by asterisks. Arrows represent probable insertion sites. The alignment was performed using the program CLUSTAL O [27].

```
Chaetomium_thermophilum_var._thermophilum_cox3-i2      TCAATTGTTATATTTTTAACAATGTAAT---TAATCGTTCTTCTTCTTCTCAA
Hypomyces_aurantius_cox3-i2_intron_with_orf330         ------TTAGTTATTCTAACAATGTTAATTATTATTCTCACTTAAAAACTAAA
                                                              *  *  ** ********* *    * **  *   *      ** **

Chaetomium_thermophilum_var._thermophilum_cox3-i2      GTAACAAAAGATTTTATTCTACTATGTCTGTTGAGCGTAAAATACTATCTCCT
Hypomyces_aurantius_cox3-i2_intron_with_orf330         GCAATAAAAGATTCTATTCTACTATACCCGAAGAGCGTAATAACCTTTCTCCT
                                                       * ** ******** ***********  * *  ******** *  ** ******

Chaetomium_thermophilum_var._thermophilum_cox3-i2      TAACAGGATTTGCGGATGCTGAATCAAGTTTTAGTCTAAAGGTATCTAAAAAA
Hypomyces_aurantius_cox3-i2_intron_with_orf330         TAACAGGATTTGCCGACGCAGAGTCAAGTTTTAGTCTAAAAGTATCTAAAAAA
                                                       ************* ** ** ** ***************** ************

Chaetomium_thermophilum_var._thermophilum_cox3-i2      CTAGATCAGGATGACATATTATTCCTGAATTTAGGATAGAGTTACATAATAGA
Hypomyces_aurantius_cox3-i2_intron_with_orf330         CTAAATCAGGGTGACACGTAATACCTGAATTTAGAATAGAGTTACACAATAGA
                                                       *** ****** *****  * ** *********** *********** ******

Chaetomium_thermophilum_var._thermophilum_cox3-i2      ATCTATTAAGAGAAATACATGCCTTTTTTGGGGTAGGTATTATCACAGAGATT
Hypomyces_aurantius_cox3-i2_intron_with_orf330         ACTTATTAAGAGAAATACACGCCTTTTTTGGGGTAGGTCATATTAGTGAGTAT
                                                       *  **************** *****************  *** *  ***  *

Chaetomium_thermophilum_var._thermophilum_cox3-i2      AAAATAAGGTAGTTTATAGTGTACAATCGCGTCGAGATTTAACTAATGCAATT
Hypomyces_aurantius_cox3-i2_intron_with_orf330         TAAATAAGGTAGTTTACAGTGTTCAATCCTCTCGTGATTTAGCTAATGTAATT
                                                        *************** ***** *****   *** ****** ******* ****

Chaetomium_thermophilum_var._thermophilum_cox3-i2      ATTTTGATCAATATCCTTTAATAACTCAAAAAAAGGCGGATTACTTTTTATTT
Hypomyces_aurantius_cox3-i2_intron_with_orf330         ATTTTGACAAATATCCTTTAATAACTCAAAAAAATGCTGATTACCTTTTATTT
                                                       *******  ************************* ** ****** ********

Chaetomium_thermophilum_var._thermophilum_cox3-i2      CTGTTGAGATGTTATGTTTAAATGCGCAATCAGATATTAGGGGAGTTCAAGAT
Hypomyces_aurantius_cox3-i2_intron_with_orf330         CTATTGAGCTATTAAATTTAAAGATACATTCAGACATTAACGGTTTTCAAGGT
                                                       ** ***** * ***  ******    ** ***** ****  **  ****** *

Chaetomium_thermophilum_var._thermophilum_cox3-i2      GTCTAAAAGCAGCAATGAATAAAGGATTGTCTGATAAATTAAATTCTGAGTTT
Hypomyces_aurantius_cox3-i2_intron_with_orf330         GACTTAAAGCAGCAATGAACTGAGGATTGTCAGACAAATTAATGAATGAGTTC
                                                       * ** **************   ********* ** *******    ******

Chaetomium_thermophilum_var._thermophilum_cox3-i2      ATGTTATCCCCGCCGAACGTCCTACAATTAATTTTGAAAGAATACCTGATCCA
Hypomyces_aurantius_cox3-i2_intron_with_orf330         GTGTTATTTCAGCTATACGTCCTGCAGTAAATTTTGAAGGTATACCCAATCCT
                                                        ******  * **   ******* ** * ********* * *****  ****

Chaetomium_thermophilum_var._thermophilum_cox3-i2      TAGCAGGTTTCGTGGACGGAGAGGGTTGTTTCTATATTAACACAAAAAAAGCT
Hypomyces_aurantius_cox3-i2_intron_with_orf330         TAGCAGGTTTCGTGGATGGAGAAGGTTGTTTTTATGTTAACACGAAAAAAGCT
                                                       **************** ***** ******** *** ******* *********

Chaetomium_thermophilum_var._thermophilum_cox3-i2      ATATAACTGGTTATCAGGTTATCATGACATTCTCTATATCTCAACATGTAAGA
Hypomyces_aurantius_cox3-i2_intron_with_orf330         ATTTAACAGGATATCAGGTTATTATGTCCTTCTCTATATGTCAACATGTAAAA
                                                       ** **** ** *********** *** * ********** *********** *

Chaetomium_thermophilum_var._thermophilum_cox3-i2      TTTTATTAAGAAAGTTTATAGATTACTTAAATTGTGGTAATATAGAAAAAGTG
Hypomyces_aurantius_cox3-i2_intron_with_orf330         TTTTATTAAATAAGTTCACAGATTACTTAGGTTGTGGTAAAATAGAAAAAGTT
                                                       *********  ***** * **********  ********* ***********

Chaetomium_thermophilum_var._thermophilum_cox3-i2      GACCTGATGGAGTTAAATTTATTGTATATAAATTTAGTGATATTAGAGACAAA
Hypomyces_aurantius_cox3-i2_intron_with_orf330         GACCTGATCAGGTAACATTTGTTACCTATAAATTTAGTGATATTCGTGATAAG
                                                       ********   ** * **** **   ***************** * ** **

Chaetomium_thermophilum_var._thermophilum_cox3-i2      CTTTTTTCCAAAATTACCCTTTACATGGAATGAAGTCTAAGGATTTTAGAGAT
Hypomyces_aurantius_cox3-i2_intron_with_orf330         CTTTTTTCCAAAACTATCCTTTACACGGGGTGAAATCTAAGGATTACAGAGAT
                                                       ************* ** ******** **  **** **********  ******

Chaetomium_thermophilum_var._thermophilum_cox3-i2      AAATAGCTAAACTTATGGAAAACAAATCACATTTAACTCTTGACGGTTTCAAG
Hypomyces_aurantius_cox3-i2_intron_with_orf330         AGATAGTTAAACTTATGGAAAATAAATCACATTTAACTCTTGACGGTCTTAAT
                                                       * **** *************** ************************ * **

Chaetomium_thermophilum_var._thermophilum_cox3-i2      AATCTTTAAAATCTGGTATGAATAGAGGAAGAATGTAGAATGCGCGATTAATT
Hypomyces_aurantius_cox3-i2_intron_with_orf330         AATCATTAAAATCTGGAATGAATAGAGGTAGAATATACGAT------------
                                                       **** *********** *********** ***** **  **

Chaetomium_thermophilum_var._thermophilum_cox3-i2      CGATTATGATGTGGAGGCCCTTTTAATTATTAATATTAATTAAATGTTAATAT
Hypomyces_aurantius_cox3-i2_intron_with_orf330         -----------------------------------------------------


Chaetomium_thermophilum_var._thermophilum_cox3-i2      TTAATAGCAAATATTGGAACCTTTATAACGTGGAGGCCCTTTTATATGATTAT
Hypomyces_aurantius_cox3-i2_intron_with_orf330         --------TAATATTGGAACCTTTATAACGTGGAGGCCCTTTTATATGATTAT
                                                                *********************************************

Chaetomium_thermophilum_var._thermophilum_cox3-i2      TAAATATTAATATTAATAATTAAATAGAAAACAAAGTTAAATGCATAGAACTT
Hypomyces_aurantius_cox3-i2_intron_with_orf330         TAAATATTAATATTAATAATTAAATAGAAAACAAAGTTAATTGCATGAAACTC
                                                       **************************************** *****  ****

Chaetomium_thermophilum_var._thermophilum_cox3-i2      -TAAGAGACAATATGCAGCCAAATATTATTAGAACATTAAAATAATTTCTAAT
Hypomyces_aurantius_cox3-i2_intron_with_orf330         TATTGAGATAATATGCAGCCAAACATTATTAGAATATTAAATTAATTTTTAAT
                                                           **** ************** ********** ****** ****** ****

Chaetomium_thermophilum_var._thermophilum_cox3-i2      GTTCAACGACTAAAAAGATAGATTTCCTATCTTGCAAATTTTGTTGTATAATA
Hypomyces_aurantius_cox3-i2_intron_with_orf330         GTTCAACGACTAAAAAGATAGATCTTCTATCTTGCAAATTTTGCTGTATAGTA

                                                       *********************** * ***************** ****** **

Chaetomium_thermophilum_var._thermophilum_cox3-i2      TATTATACAAGTGACATAGTCTGAACAATAATGAAAATTATTGAAGGATTGAT
Hypomyces_aurantius_cox3-i2_intron_with_orf330         TATTATATGGATGATATAGTCTGAACAATAATTAAAATTATTGAAGGATTTAA
                                                       *******    *** **************** ***************** *

Chaetomium_thermophilum_var._thermophilum_cox3-i2      TGCGCATTAAATCCGTTAACAAATTTTTTTTTG--------------------
Hypomyces_aurantius_cox3-i2_intron_with_orf330         ATCCGTTAACAAAAAAAAAAAAGATTAGCCTTGTTTTCTTATTAGCTTTCATGG
                                                         *   * * *      ** * ***    **
```

Figure S2. Alignment between cox3-i2 of *Chaetomium thermophilum* var. thermophilum and upstream intron in cox3-i2 of Hypomyces aurantius. Among 1339 base pairs compared, 1069 were identify (79.8%). Consensus sequences were marked by asterisks. Gaps were indicated by dash lines. Comparison were performed by CLUSTAL O 1.2.1.

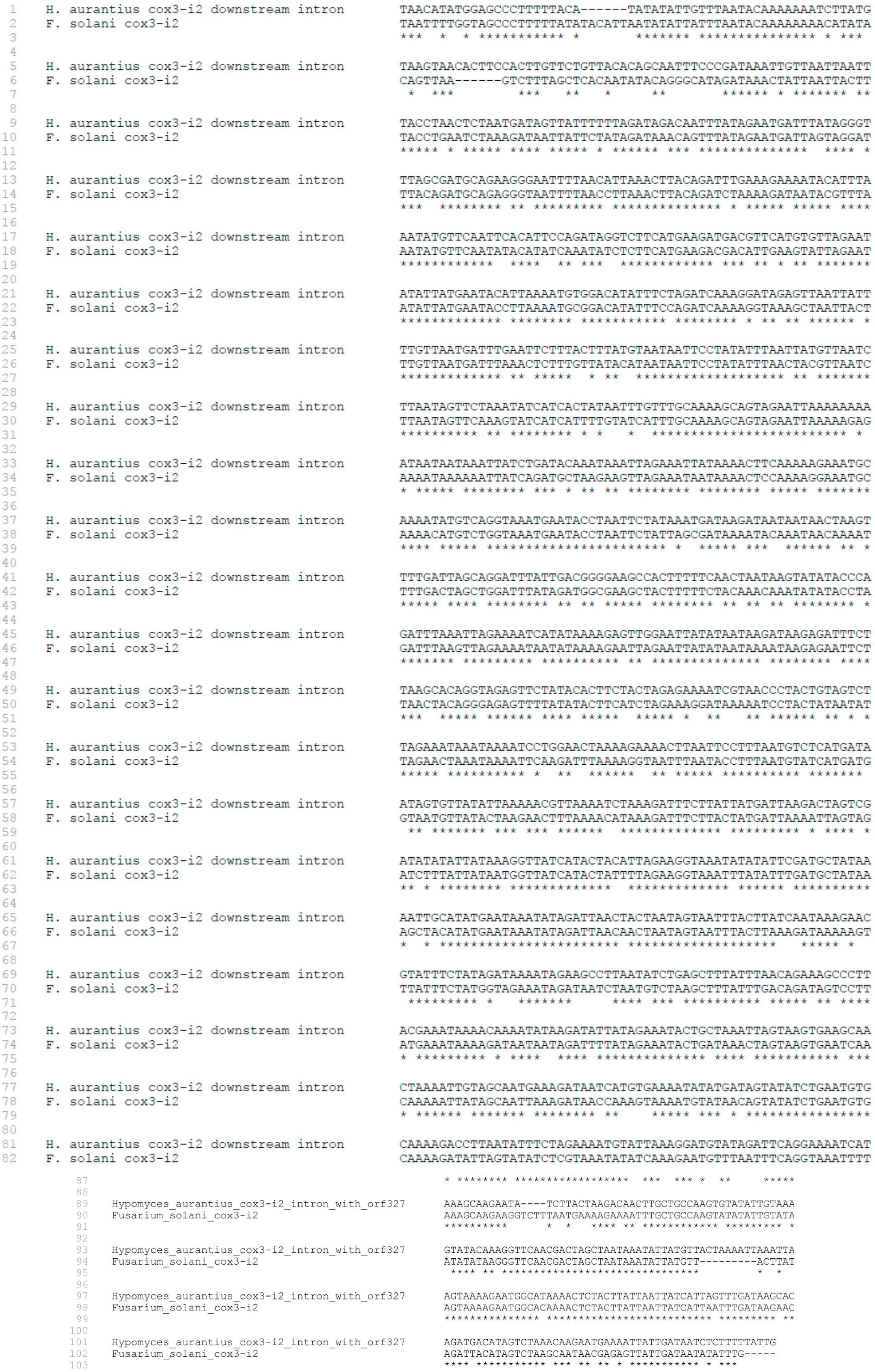

Figure S3. Alignment between cox3-i2 of *Fusarium solani* and downstream intron in cox3-i2 of Hypomyces aurantius. Among 1538 base pairs compared, 1234 were identify (80.2%). Consensus sequences were marked by asterisks. Gaps were indicated by dash lines. Comparison were performed by CLUSTAL O 1.2.1.

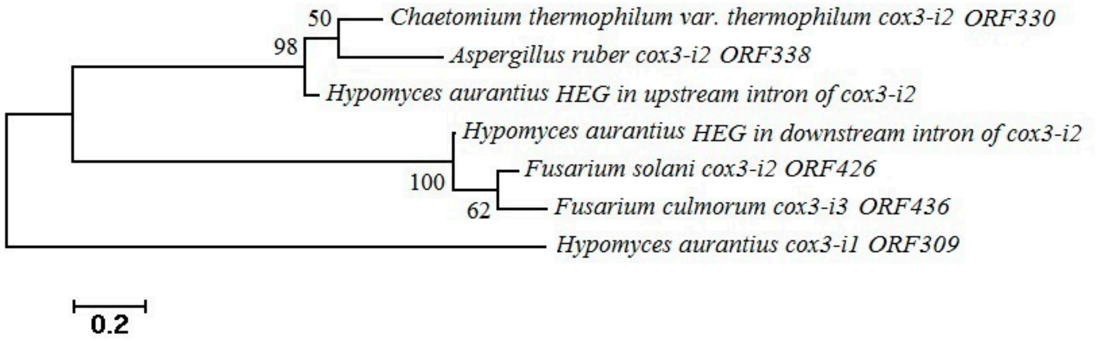

Figure S4. Phylogenetic analysis for translated protein sequences of six ORFs/HEGs. HEG in upstream intron of cox3-i2 showed highest similarity with ORF330 in cox3-i2 of *C. themophilum* var. thermophilum and ORF338 in cox3-i2 of A. ruber, and HEG in downstream intron matched well with ORF426 in cox3-i2 of *F. solani* and ORF436 in cox3-i3 of *F. culmorum.* ORF309 in cox3-i1 (group IB) of *H. aurantius* was used as an outgroup. Comparing with the ORF309, the relationship between two HEGs in cox3-i2 of *H. aurantius* was much closer.

Table S1. Conserved protein-coding genes and ribosomal RNA subunits in *H. aurantius* mitochondrial genome.

Gene	Start	End	Strand	Gene Size, bp	CDSSize, bp	Intron Number	Average Intron Length, bp	Percent of Intronic Sequences, %	Stop Codon
rns	1	1505	+	1505				0	
cox3	3516	8330	+	4815	810	2	2003	83.2	TAA
nad6	8565	9242	+	678	678			0	TAA
rnl	11,589	22,088	+	10,500		4	1993	80	
rps3	17,762	19,186	+	1425	1425			0	TAG
nad2	30,234	31,901	+	1668	1668			0	TAA
nad3	33,265	33,678	+	414	414			0	TAA
atp9	33,856	34,050	+	195	195			0	TAG
cox2	35,317	39,361	+	4045	750	1	1795	81.5	TAA
nad4L	41,027	41,296	+	270	270			0	TAA
nad5 [a]	41,296	44,055	+	2760	2760			0	TAA
cob	44,271	49,297	+	5027	1170	3	1286	76.7	TAA
cox1	49,947	60,590	+	10,644	1587	5	1811	85.1	TAA
nad1	61,752	62,852	+	1101	1101			0	TAA
nad4	63,999	65,456	+	1458	1458			0	TAA
atp8	66,581	66,727	+	147	147			0	TAA
atp6	66,860	70,630	+	3771	783	2	711	79.2	TAA

[a] *Nad*5 gene uses the last A of nad4L termination codon as first nt, blank space means no present or observed, or unable to calculate.

Table S2. The frequency of utilization of the 64 standard codons.

Amino acid	Codon	Frequency	Amino acid	codon	Frequency	Amino acid	codon	Frequency	Amino acid	codon	Frequency
Phe	UUU	571	Ser	UCU	426	Tyr	UAU	568	Cys	UGU	87
	UUC	256		UCC	48		UAC	165		UGC	19
Leu	UUA	1158		UCA	269	TER	UAA	34	Trp	UGA	130
	UUG	104		UCG	27		UAG	10		UGG	11
	CUU	201	Pro	CCU	254	His	CAU	200	Arg	CGU	93
	CUC	8		CCC	22		CAC	69		CGC	4
	CUA	139		CCA	91	Gln	CAA	264		CGA	3
	CUG	29		CCG	16		CAG	35		CGG	1
Ile	AUU	501	Thr	ACU	350	Asn	AAU	836	Ser	AGU	387
	AUC	117		ACC	24		AAC	216		AGC	73
	AUA	791		ACA	339	Lys	AAA	955	Arg	AGA	372
Met	AUG	277		ACG	20		AAG	153		AGG	18
Val	GUU	307	Ala	GCU	328	Asp	GAU	448	Gly	GGU	380
	GUC	32		GCC	72		GAC	73		GGC	24
	GUA	367		GCA	186	Glu	GAA	537		GGA	279
	GUG	68		GCG	31		GAG	136		GGG	65

14,074 codons in Average of genes (used Filamentous fungi Mito).
